# The prevalence and types of discordance between physician perception and objective data from standardized measures of rheumatoid arthritis disease activity in real-world clinical practice in the US

**DOI:** 10.1186/s41927-019-0073-8

**Published:** 2019-07-04

**Authors:** Wenhui Wei, Emma Sullivan, Stuart Blackburn, Chieh-I Chen, James Piercy, Jeffrey R. Curtis

**Affiliations:** 1 Regeneron Pharmaceuticals, Inc., 777 Old Saw Mill River Road, Tarrytown, NY USA; 2 Adelphi Real World, Manchester, UK; 3 University of Alabama at Birmingham, Birmingham, AL USA

**Keywords:** Rheumatoid arthritis, Rheumatologists’ evaluation, Disease activity, Remission, Pain, Joint damage

## Abstract

**Background:**

Heterogeneity in assessments of rheumatoid arthritis (RA) disease remission, based on physician judgment and patient self-reports versus standardized measures, have previously been reported. This study explored the prevalence and types of discordance between physician perception versus objective data of RA disease activity in real-world clinical practice in the US.

**Methods:**

Data were from the Adelphi RA Disease Specific Programme (DSP; January to March 2014), a cross-sectional survey of US rheumatologists and their patients. RA remission based on physician judgment versus Disease Activity Score in 28 joints (3)-erythrocyte sedimentation rate (DAS28(3)-ESR) and Clinical Disease Activity Index (CDAI) scores were compared using descriptive analyses; patient and physician factors associated with discordance were identified using bivariate and multivariate analyses.

**Results:**

Of 101 rheumatologists participating (completing patient-record forms for 843 patients), 56.4% based assessment of remission on clinical judgment alone. Of 531 patients eligible for the discordance analysis, 49.7% were in remission based on rheumatologists’ evaluation, and 30.7% were eligible based on DAS28(3)-ESR. Compared with DAS28(3)-ESR criteria, 25.8% of patients’ disease remission was negatively discordant (overestimated remission) based on clinical perception. These patients were mostly administered biologic disease-modifying antirheumatic drugs and were without a treat-to-target strategy followed by their rheumatologist (*P* < 0.05). These patients were also more likely to have experienced a higher level of pain as well as increased joint inflammation and damage (e.g. destruction of cartilage, thinning of bone, and/or synovium inflammation) compared with concordant patients (*P* < 0.005). Conversely, 6.8% of rheumatologists were positively discordant (under estimated remission) versus the DAS28(3)-ESR. Sensitivity analysis indicated different levels of discordance using CDAI, with 35.6% negative discordance and 1.3% positive discordance of rheumatologist-assessed disease remission compared with objective data.

**Conclusion:**

There is discordance between RA remission as assessed by rheumatologist perception versus standardized measures among those in the US DSP sample. Our study identified the factors associated with the discordance which may inform strategies to enhance assessments of RA disease remission.

**Electronic supplementary material:**

The online version of this article (10.1186/s41927-019-0073-8) contains supplementary material, which is available to authorized users.

## Background

The aim of rheumatoid arthritis (RA) treatment is to focus on the achievement of disease remission, preventing or halting joint damage and subsequent functional impairment [1–6]. The use of standardized measures to evaluate RA disease activity is warranted, with six such measures recommended by the American College of Rheumatology (ACR) for use during clinical evaluations. These include three patient-reported assessments: Patient Activity Scale (PAS), PAS-II, and the Routine Assessment of Patient Index Data (RAPID-3). Clinically-reported assessments include the Clinical Disease Activity Index (CDAI) and two other composite measures based on laboratory acute-phase reactants – the Simplified Disease Activity Index (SDAI) and Disease Activity Score in 28 joints (DAS28) [7, 8].

Similarly, the European League Against Rheumatism (EULAR) also recommends routine quantitative measurement of disease activity, coupled with a treat-to-target (T2T) strategy to improve patient outcomes. In this approach, treatment decision-making for the objective of achieving low disease activity or remission is made in collaboration with the patient; a variety of instruments to assess disease activity over time are available to facilitate this endeavor (2014 T2T guidelines were the latest at the time of this study; however, newer guidelines are now available) [9, 10].

Despite recommendations for their use, no such single measure has been established as a “gold standard”. In the US alone, the proportion of rheumatologists who use any standardized measure to assess RA disease activity remains low [7, 8, 10–12]. In 2014, only 3.5, 16.2, 16.5, and 26.7% of US rheumatologists used the SDAI, CDAI, DAS28, or the RAPID-3, respectively; moreover, up to 55% of rheumatologists did not administer any formal measure of RA disease activity, as indicated by a 10 year longitudinal evaluation [12]. Separately, a large level of heterogeneity in remission as per physician judgment and patient self-reports, between the types of reports or versus standardized measurements of disease activity, has also been observed in the literature [13–20]. Hence, despite the growing increase in use and evidence supporting the clinical need of standardized disease remission assessment, a gap remains between the guidelines and real-world practice, potentially limiting optimal treatment and patient outcomes [11, 18]. Therefore, establishing the magnitude and likely factors of the discordance is critical.

This study explored the prevalence and types of discordance between RA disease remission assessed on physician perception versus objective data from standardized measures as occurring in US real-world clinical practice. Patient factors including demographics and clinical status associated with the discordance were also identified.

## Methods

### Study design

Data were drawn and anonymised from the Adelphi Real-World RA Disease Specific Programme (DSP), a cross-sectional, geographically diverse, real-world survey of rheumatologists and their patients with RA [21].

Participants of the DSP comprised US rheumatologists meeting the DSP inclusion criteria (described below) who completed a survey about their attitudes and stated behaviors regarding the treatment of their RA patients. Following the survey, rheumatologists then completed patient record forms for at least the eight consecutive RA patients who attended an appointment at their clinic. The patient forms collected information on patient demographics, symptomology (including marginal bone erosion and synovium inflammation), disease severity, treatment history and concomitant conditions. While symptomology, including osteoporosis and non-RA-related bone/joint inflammation was recorded, the rheumatologists were not asked to indicate how the symptoms were assessed. In addition, rheumatologists were not required to calculate or consult any composite scores (e.g. DAS28(3)-ESR, RAPID-3, etc.) specifically for the DSP research. Test results and disease activity scores may have been separately obtained as part of the routine clinical work-up. Rheumatologists also indicated their own satisfaction with the patient’s RA control. Data collection was conducted between January and March 2014. All participating rheumatologists were compensated according to fair market research rates, reflecting the time needed to complete all the forms.

### Inclusion criteria

Rheumatologists were considered eligible to participate in the DSP if they met the following self-reported criteria: consultations with and medical management of ≥ 8 patients with RA per month, and graduation from medical school between 1975 and 2010.

Patients were considered eligible for inclusion of their data in the DSP if the following criteria were met: the patient aged ≥ 18 years, had rheumatologist confirmed and documented diagnosis of RA, and was not currently enrolled in a clinical trial. Finally, only patients for whom a DAS28(3)-ESR and CDAI score could be calculated, for purposes of the primary and sensitivity analyses, respectively, were included in the analysis population.

### Patient demographics and baseline clinical characteristics

Data on patient demographics and baseline clinical characteristics were obtained. These included the following variables: Age, sex, bodymass index (BMI), race, time since diagnosis of RA, worst ever pain experienced, current level of pain, mean, current DAS28(3) score, presence of marginal bone erosion, synovium inflammation, osteoporosis present, RA-related bone/joint inflammation or damage present, whether on biologic biologic disease modifying antirheumatic drug (bDMARD) treatment, managed by physician based in hospital or mixed (hospital + office) practice, rheumatologist has an agreed T2T measure for patient, and the length of time managed by current rheumatologist.

### Outcome measures

To evaluate rheumatologist-reported use of standardized disease activity measures, each rheumatologist was asked how he or she determined RA remission and which standard measure (if any) was typically used in assessing RA disease activity. In each patient record, physicians also stated whether the DAS28, ACR/EULAR, RAPID-3, and/or Health Assessment Questionnaire-Disability Index (HAQ-DI) assessment(s) were completed for the patient during the reference consultation.

RA disease remission for each patient was assessed via a direct question “Is this patient currently in remission? Yes or no?” (i.e. rheumatologist-reported assessment), via a calculation of RA disease activity using standardized measures, and based on information provided by rheumatologists on the record forms.

### DAS28(3)-ESR (primary analysis)

Primary analysis was conducted using DAS28(3)-ESR as this maximized the number of patients and is one of a selection of standardized measures advocated by the ACR [7, 8].

The most recent Tender Joint Count (TJC), Swollen Joint Count (SJC), and ESR values were used to calculate the DAS28(3)-ESR based on the published scoring equations [22]. Two outcome categories were defined: remission (DAS28(3)-ESR < 2.6) and no remission (DAS28(3)-ESR ≥ 2.6) [23].

### CDAI (sensitivity analysis)

CDAI was included in the sensitivity analysis as an alternative disease activity measure that does not require measurement of an acute phase reactant [24], therefore affording it a greater feasibility for implementing it in clinical practice. CDAI was calculated for patients for whom data on TJC, SJC, Evaluator’s (rheumatologist) Global Assessment (EGA) of disease activity based on a visual analog scale (0–10 cm), and Patient Global Assessment (PGA) measures had been provided on the patient forms, and scored via published equations [25]. Two outcome categories were similarly defined on this measure: remission, (CDAI ≤ 2.8) and no remission (CDAI > 2.8) [26].

### Remission discordance/concordance

The outcomes of the clinical assessment as based on physician judgment versus the standardized measures were then compared to create four groups for analysis purposes:

1. Concordant/in remission: patient in remission as per physician judgment, confirmed by standardized measure.
2. Concordant/not in remission: patient not in remission as per physician judgment, confirmed by standardized measure.
3. Rheumatologist-negative discordance: patient in remission as per physician judgment, but has active disease per standardized measure (i.e. rheumatologist underestimated disease activity versus standardized measure).
4. Rheumatologist-positive discordance: patient not in remission as per physician judgment, but standardized measure indicates no disease activity i.e. in remission (i.e. rheumatologist overestimated disease activity versus standardized measure).

### Statistical analysis

All analyses were conducted with Stata^®^ 14.0 or later (StataCorp LLC, College Station, TX, US). Descriptive analyses provided the frequency (n) and percentage (%) of rheumatologist self-reported use of standardized measures and of patients assigned to each of the four cohorts, including respective rheumatologist and patient characteristics. Bivariate and multivariate analyses were conducted to identify factors associated with Rheumatologist-negative discordance versus the Concordant/in remission cohort. Odds ratios (OR), 95% confidence interval (CI), and *P* values indicated the robustness and significance of the results. The differences between cohorts were examined across various patient characteristics, including patient demographics, disease status, RA symptoms, treatment, doctor-patient relationship, patient-reported data including health-related quality of life, and rheumatologist self-reported characteristics such as workload and practice setting. Fisher Exact test, Mann-Whitney test, or Pearson’s chi-squared test assessed significant differences between patient subgroups on binary, non-parametric, and categorical outcomes, respectively. Additionally, Kernel density estimations using the Gaussian Kernel function were calculated and plotted for DAS28(3)-ESR for the four concordance groups.

Multivariate analyses then identified patient and physician characteristics independently associated with Rheumatologist-negative discordance of remission. Variables hypothesized to be associated with negative discordance were selected for inclusion in a logistic regression model. Variable selection was guided by disease knowledge; variables included physician practice type, whether a T2T management approach was in place for the patient, time since patient diagnosis, change in patient pain (from worse ever to current pain^1^), and presence of RA-related bone or joint inflammation or damage.

Standard errors were adjusted in the regressions to model the intragroup correlation (or clustering) of patients within rheumatologist practice using the Huber and White sandwich estimator of variances [27]. A 95% significance level was used throughout.

## Results

### Remission assessment

#### Rheumatologist self-reported use of standardized measures

The 2014 DSP survey included 101 US rheumatologists who provided 843 RA patient records for the analysis (Table 1). The majority of the rheumatologists were male (72.3%) and located in the East (36.6%) or Midwest (28.7%) of the US; 38.6% reported an office-based practice.

**Table 1:** Patient demographics and clinical characteristics

Characteristic	Concordant			Discordant	
	Overall (*n* = 531)	Not in remission (*n* = 231)	In remission (*n* = 127)	Rheumatologist negatively discordant^a^ (*n* = 137)	Rheumatologist positively discordant (*n* = 36)
Age, in years, mean (SD)	56.4 (15.5)	56.7 (15.5)	53.1 (16.6)	59.0 (14.2)**	56.4 (15.2)
Female gender, n (%)	397 (74.8)	177 (76.6)	89 (70.1)	101 (73.7)	30 (83.3)
BMI, mean (SD)	28.0 (6.3)	28.3 (6.9)	27.3 (5.9)	27.7 (5.3)	29.0 (6.8)
White, n (%)	363 (68.4)	152 (65.8)	87 (68.5)	94 (68.8)	30 (83.3)
Time since diagnosis of RA, years (SD)	7.6 (8.1)	7.2 (8.6)	5.9 (6.1)	9.8 (8.8)**	7.7 (7.3)
Current assessment of level of pain, mean (SD)^b^	2.8 (2.0)	4.2 (2.2)	1.4 (0.9)	1.9 (1.0)	2.4 (1.2)
Current DAS28(3) score, mean (SD)	3.6 (1.5)	1.9 (0.6)	4.8 (1.2)	1.9 (0.5)	3.7 (0.8)
Marginal bone erosion present, n (%)	164 (30.9)	89 (38.5)	20 (15.7)	48 (35.0)**	7 (19.4)
Synovium inflammation present, n (%)	185 (34.8)	132 (57.1)	9 (7.1)	32 (23.4)**	12 (33.3)
Osteoporosis present, n (%)	127 (23.9)	68 (29.4)	19 (15.0)	37 (27.0)*	3 (8.3)
No RA-related bone/joint inflammation or damage present, n (%)	179 (33.7)	44 (19.0)	75 (59.1)	47 (34.3)**	13 (36.1)
On bDMARD, n (%)	283 (53.3)	120 (51.9)	58 (45.7)	83 (60.6)*	22 (61.1)
Patient managed by physician based in hospital or mixed (hospital + office) practice, n (%)	332 (62.5)	138 (59.7)	92 (72.4)	80 (58.4)*	22 (61.1)
Rheumatologist has an agreed T2T measure for patient, n (%)	213 (40.1)	81 (35.1)	68 (53.5)	54 (39.4)*	10 (27.8)
Time managed by current rheumatologist, years (SD)	4.5 (4.6)	4.0 (4.9)	4.4 (4.1)	5.3 (4.0)**	5.1 (5.8)

*bDMARD* Biologic disease modifying antirheumatic drug, *BMI* Body mass index, *DAS28(3)* Disease activity score in 28 joints 3, *RA* Rheumatoid arthritis, *SD* Standard deviation, *T2T* Treat-to-target

*P < 0.05

**P < 0.005

^a^Bivariate analysis was performed between Concordant – in remission and Rheumatologist – negatively discordant cohort. Rheumatologist – negatively discordant: patient in remission as per rheumatologist evaluation, but not in remission by standardized measure (i.e. rheumatologist underestimating disease activity versus standardized measure). Rheumatologist – positively discordant: patient not in remission as per rheumatologist evaluation, but in remission by standardized measure. (i.e. rheumatologist overestimating disease activity versus standardized measure)

^b^Low [1–3], Medium/high [4–10]

**Fig. 1:**
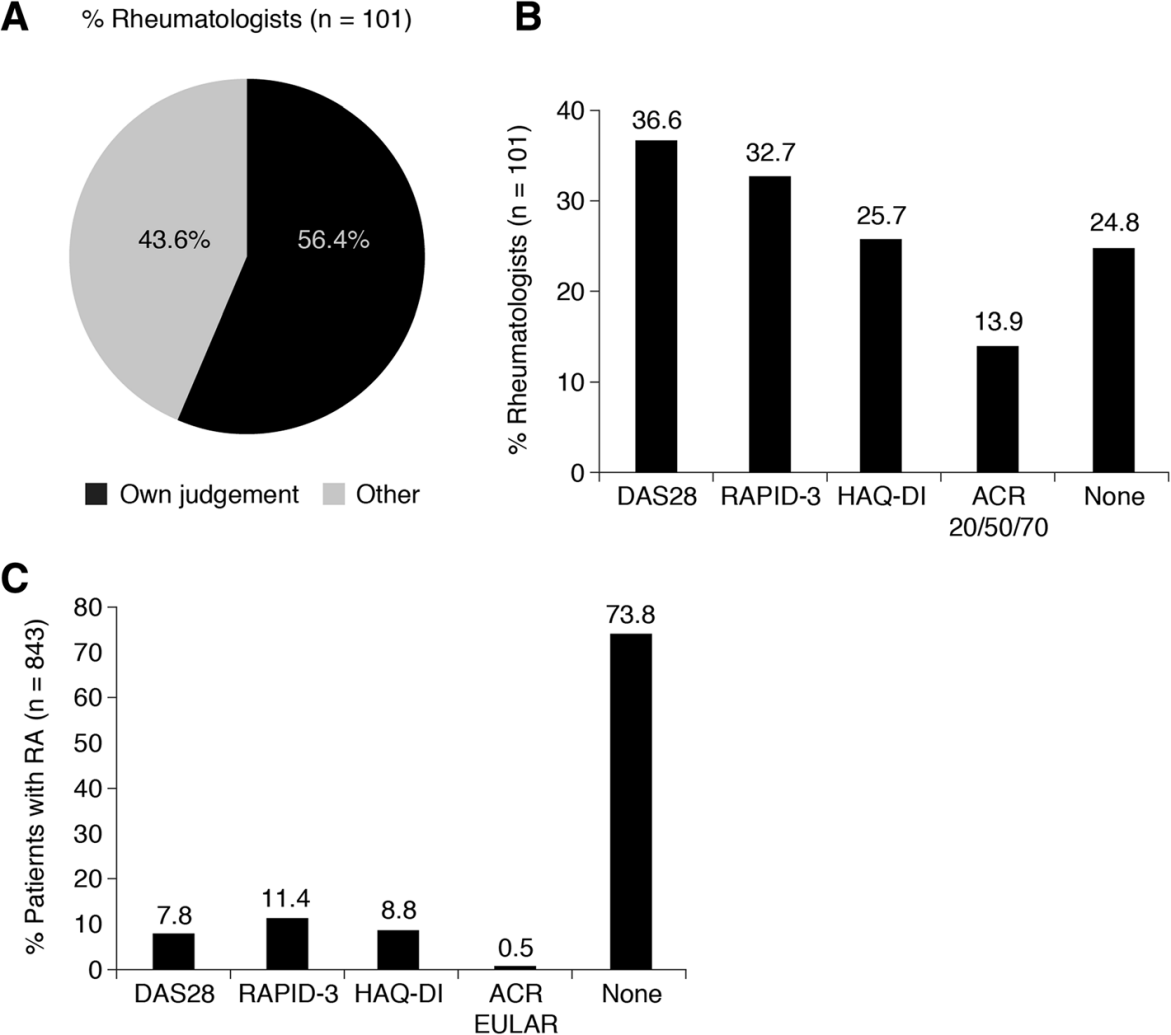
Rheumatologist assessment of patient remission. **a** Rheumatologist reported method of assessing remission of patient. **b** Typical measures used by rheumatologists to assess RA disease activity. More than one measure could be selected. **c** Use of standardized measures during the patient’s current visit. *ACR* American College of Rheumatology; *DAS28* Disease Activity Score in 28 Joints; *EULAR* European League Against Rheumatism; *HAQ-DI* Health Assessment Questionnaire-Disability Index; RA rheumatoid arthritis; *RAPID* Routine Assessment of Patient Index Data. Rheumatologists could state that they had completed none of the assessments or select as many as they completed

Overall, 56.4% of rheumatologists reported using only clinical judgment to assess RA disease remission in their patients (Fig. 1a). The most widely used standardized measures of RA disease activity used by the participating rheumatologists were the DAS28 (36.6%) and the RAPID-3 (32.7%) (Fig. 1b). However, at the “current” visit (i.e. the visit during which the rheumatologist completed the patient form), rheumatologists used either one or more of the DAS28, ACR/EULAR, RAPID-3, and/or HAQ-DI criteria to assess RA disease activity in only one in four patients overall (Fig. 1c), while the remaining patients were not assessed by standardized measure.

#### Concordance between remission assessment according to Rheumatologists’ evaluation versus DAS28(3)-ESR measure

**Fig. 2:**
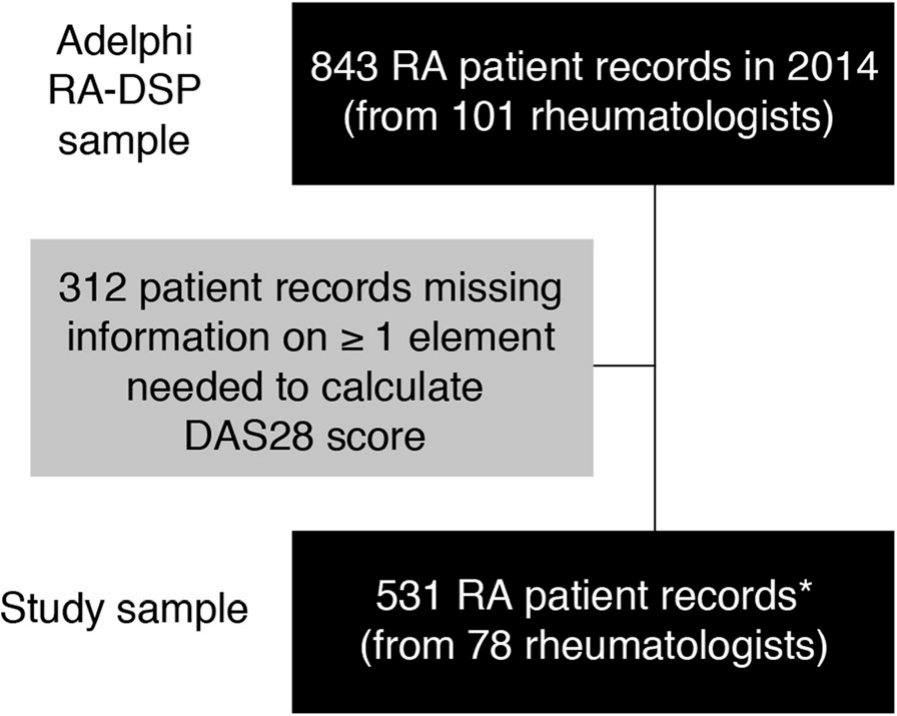
Patient attrition. *256 patients voluntarily provided self-completion forms. *DAS28* Disease Activity Score in 28 Joints, *DSP* Disease Specific Programme, *RA* rheumatoid arthritis

A total of 531 RA patient records from 78 rheumatologists (23 rheumatologists did not provide patients meeting the inclusion criteria and were therefore excluded from the analysis) had the requisite information to calculate the DAS28(3)-ESR score (Fig. 2). The mean age of the patient analysis population was 56.4 years, 74.8% were female, and the mean BMI was 28.0 kg/m^2^. More than half of the patients received bDMARD therapy (53.3%). These demographics were not statistically different in 312 patients who were excluded due to missing data on the TJC (*n* = 208; 24.7%), SJC (*n* = 198; 23.5%), and/or ESR (*n* = 172; 20.4%) measures. Data were missing in 14.0% of patients across one of these variables, in 14.5% across two, and in 8.5% across all three variables.

In total, 67.4% (*n* = 358) of cases revealed concordance between the physician perception and data from standardized measure; 23.9% (*n* = 127) of the cases were classified as Concordant/in remission, while 43.5% (*n* = 231) were classified as Concordant/not in remission (Table 2). However, in 32.6% (*n* = 173) of cases, there was discordance between the assessment according to rheumatologists’ evaluation and DAS28(3)-ESR-assessed RA disease remission; 25.8% (*n* = 137) of cases were classified as Rheumatologist-negative discordance, while 6.8% (*n* = 36) were classified as Rheumatologist-positively discordance.

**Table 2:** Concordance and discordance of rheumatologists’ evaluation versus DAS28(3)-ESR or CDAI-measured remission assessment

		Objective measure					
		DAS28(3)-ESR (*n* = 531)			CDAI (*n* = 298)		
		Total	Remission	No remission	Total	Remission	No remission
Rheumatologists’ evaluation	Total	531 (100.0%)	163 (30.7%)	368 (69.3%)	298 (100.0%)	44 (14.8%)	254 (85.2%)
	Remission	264 (49.7%)	Concordant – both remission 127 (23.9%)	Rheumatologist –negatively discordant 137 (25.8%)	146 (49.0%)	Concordant – both remission 40 (13.4%)	Rheumatologist –negatively discordant 106 (35.6%)
	No remission	267 (50.3%)	Rheumatologist – positively discordant 36 (6.8%)	Concordant – both not remission 231 (43.5%)	152 (51.0%)	Rheumatologist – positively discordant 4 (1.3%)	Concordant – both not remission 148 (49.7%)

Percentages are calculated using the total number of patients assessed based on rheumatologists’ evaluation or DAS28(3)-ESR/CDAI scores. *CDAI *Clinical Disease Activity Index, *DAS28(3)-ESR* Disease Activity Score in 28 joints (3)-erythrocyte 471 sedimentation rate

**Fig. 3:**
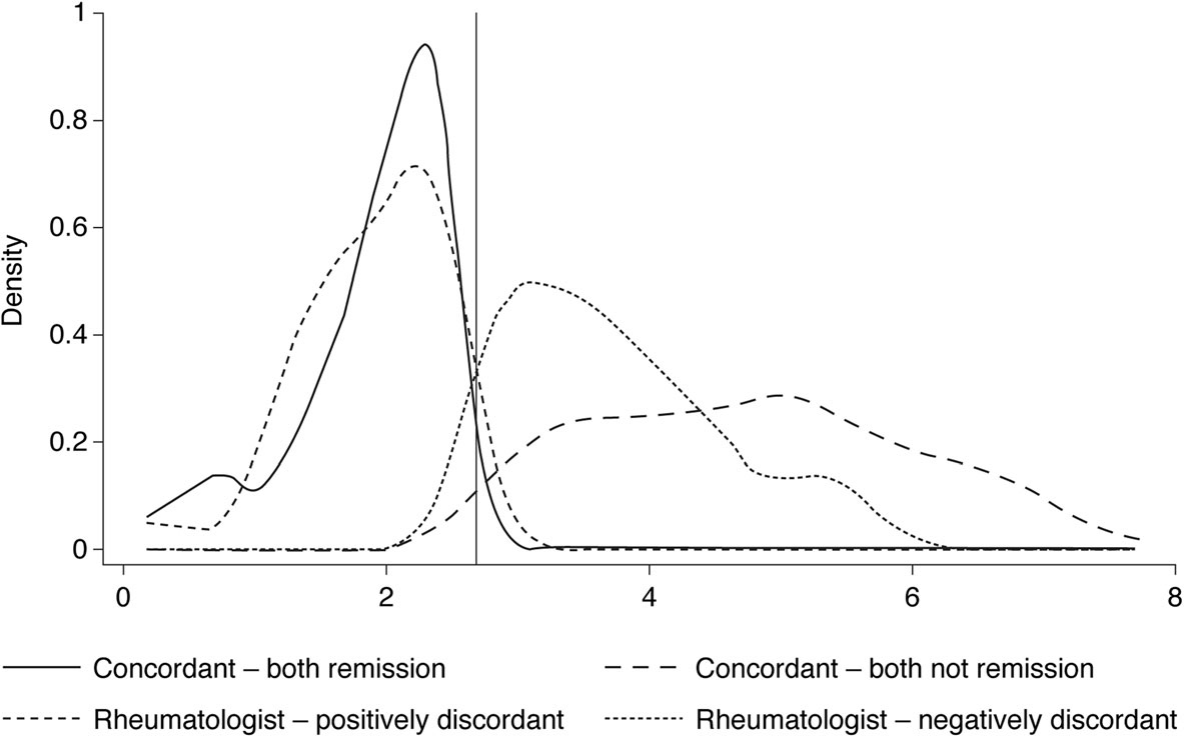
Concordance of rheumatologists’ evaluation versus DAS28(3)-ESR-measured remission assessment. Kernel density estimation of DAS28(3)-ESR by concordance group. The solid vertical line represents the remission cut-off at DAS28(3)-ESR = 2.6. Data for patients ‘in remission’ and ‘not in remission’ that appear to be falling on the right- and left-hand side of the remission cut-off, respectively, is due to the smoothing of the curve. *DAS28(3)-ESR* Disease Activity Score in 28 joints (3)-erythrocyte sedimentation rate

The DAS28(3)-ESR distribution of each discordance/concordance cohort is shown in the Kernel density plot (Fig. 3). For cases classified under Rheumatologist-negative discordance, the distribution of the DAS28(3)-ESR scores showed that the majority were under the 3.2 cut-off for low disease activity [32].

CDAI was calculated for 298 RA patient records (from 58 rheumatologists). Patients excluded from the CDAI sensitivity analysis were due to missing data on PGA (*n* = 435; 51.6%), TJC (*n* = 208; 24.7%), and/or SJC (*n* = 198; 23.5%) measures; there were no missing data for EGA. Concordant/in remission versus the CDAI was observed in 13.4% (*n* = 40) of cases and 49.7% (*n* = 148) were classified as Concordant/not in remission versus the CDAI. There were 36.9% of cases classified as discordant between rheumatologist assessment versus the CDAI (35.6% Rheumatologist-positive discordance and 1.3% Rheumatologist-negative discordance).

#### Patient and Rheumatologist characteristics associated with rheumatologist-negative discordance of RA disease remission versus concordance/in remission cohorts

The size of the Rheumatologist-positive discordance cohort was too small for statistical analysis (*n* = 36, 6.8% of overall population) and was therefore excluded from bivariate and multivariate analyses. Patient characteristics and rheumatologist factors were compared between the Rheumatologist-negative discordance and the Concordant/in remission cohorts using bivariate analysis (Table 2).

Compared with the Concordant/in remission cohort, patients in the Rheumatologist-negative discordance cohort were older, had a longer duration of RA diagnosis, and had been managed longer by their current rheumatologist (*P* < 0.005); a larger proportion had been treated in an office only setting versus a hospital and/or office setting (*P* < 0.05). These patients were more likely to have experienced a higher level of pain and increased joint inflammation and damage (e.g. destruction of cartilage, thinning of bone, and/or synovium inflammation) (*P* < 0.005). More patients were administered bDMARDs, and more patients did not have a T2T strategy in place (*P* < 0.05). There was no statistically significant difference between the Rheumatologist-negative discordance cohort and the Concordant/in remission cohorts in terms of patient BMI (mean [SD] 27.7 [5.3] and 27.3 [5.9]) in the last 12 months.

**Fig. 4:**
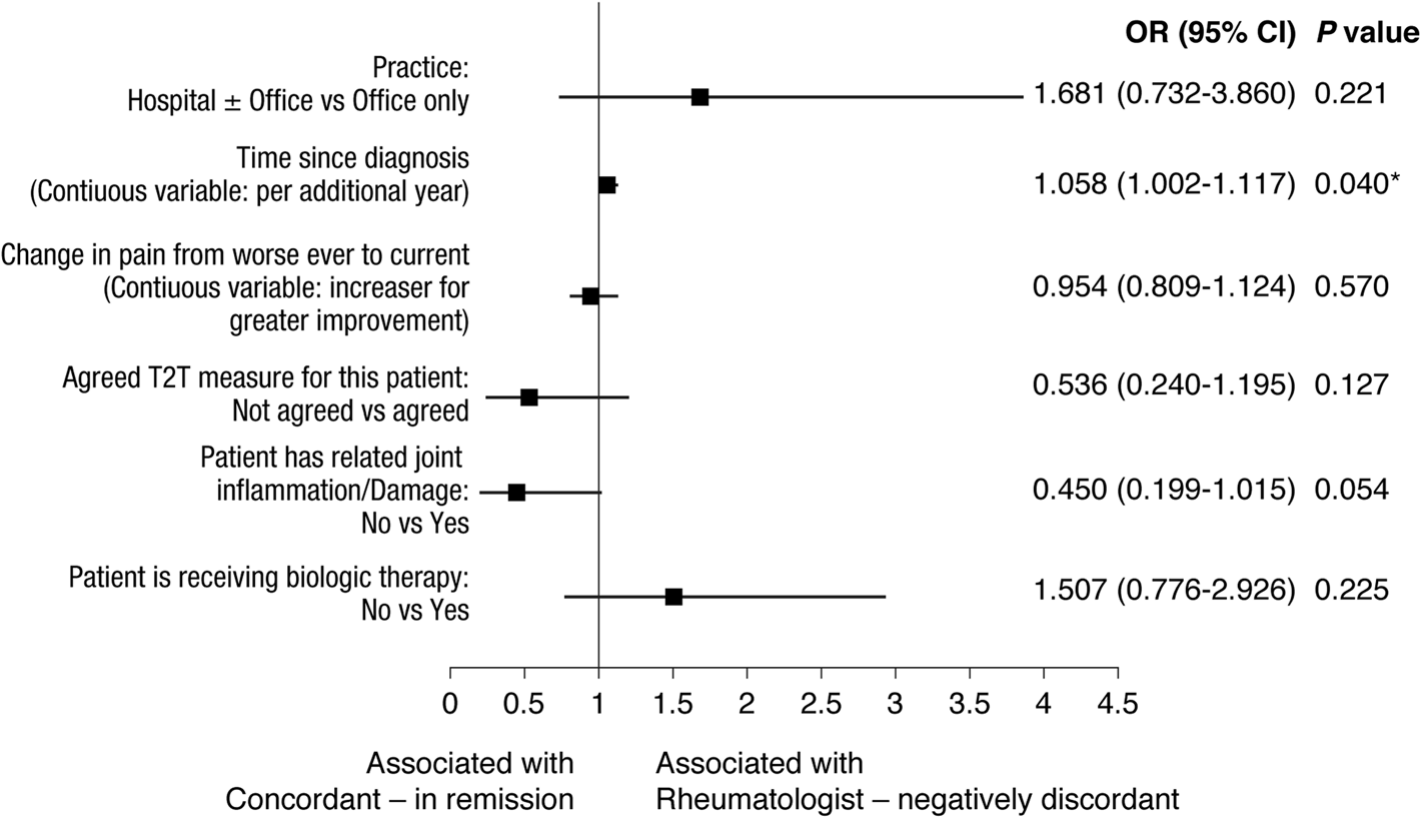
Multivariable-adjusted rheumatologist factors associated with concordance of rheumatologists’ evaluation with DAS28(3)-ESR-measured and rheumatologist negative discordance remission. *P < 0.05. *CI* Confidence interval; *OR* Odds ratio; *RA* Rheumatoid arthritis; *T2T* treat-to-target

Duration of RA diagnosis was independently associated with Rheumatologist-negative discordance outcome in both the multivariate and CDAI sensitivity analysis (Fig. 4, sensitivity analysis: OR 1.12 [95% CI 1.024–1.220], *P* = 0.013). The absence of joint inflammation or damage was suggested by the multivariate and CDAI sensitivity analysis to be associated with Concordant/in remission as based on DAS28(3)-ESR-measured remission, although it was not statistically significant (multivariate analysis: OR 0.450 [95% CI 0.199–1.015], *P* = 0.054; CDAI sensitivity analysis: OR 0.414 [95% CI 0.135–1.277], *P* = 0.125).

## Discussion

This study which was conducted in the real-world US practice setting compared the assessment of RA disease remission based on physician judgment versus standardized RA measures; we also identified patient characteristics and rheumatologist factors associated with discordance between the approaches to evaluate disease remission. Results revealed that standardized measures for the assessment of RA disease activity are not commonly or consistently used; 56.4% of sampled rheumatologists relied on their clinical judgment to determine remission, and only 26.2% of rheumatologists relied on a composite disease activity score calculated during the previous patient consultation. Our results are consistent with other studies demonstrating the limited use of standardized RA disease activity measures [12, 28].

Negative discordance was higher for the CDAI (35.6%) than the DAS28(3)-ESR (25.8%); this was expected due to the inclusion of EGA and PGA in the CDAI. In the DAS28(3)-ESR analysis, PGA was excluded to mitigate a limitation observed in previous studies [29]. In these studies, it has been proposed that PGA, perhaps associated with concomitant fibromyalgia, might drive discordance between rheumatologist judgment versus the DAS28 evaluation of remission [18, 19, 30, 31].

We also investigated patient and physician factors associated with negative and positive discordance of remission as these findings may help identify patients who are perhaps under- or over-treated. Clinical factors associated with negative discordance were expected to be different from those with positive discordance. However, cohort size for those classified as Rheumatologist- positive discordance (physician overestimation of disease activity) was limited, and therefore robust comparisons were not feasible. In itself this finding suggests that physicians are mostly negatively discordant, typically underestimating disease activity in patients under their management.

Longer time since diagnosis was associated with higher negative discordance of remission by rheumatologists. Although the reason for this association cannot be derived from this study, some explanation could be derived from a previous study showing that “remission” is a more likely treatment target in the early stages of disease (diagnosis < 2 years); targets such as reduced disease activity or symptom improvement were observed to pragmatically change with longer disease duration [32].

Standardized tools such as DAS28(3)-ESR and CDAI require rheumatologists to perform detailed, quantitative joint counts to assess RA remission. As expected, rheumatologists’ qualitative judgment regarding the absence of joint inflammation or damage was associated with concordance with DAS28(3)-ESR-measured remission. Factors associated with negative discordance included practicing in a hospital, patients having longer RA disease duration, and being in receipt of biologic therapy. Increasing duration of RA may be a proxy for greater damage and more difficulty in clinically assessing active disease.

Some potential limitations of the data derived from the Adelphi RA DSP must be noted. The rheumatologist sample may not be generalizable to rheumatologists across the whole spectrum of real-world clinical practice in the US, as the sample may be influenced by willingness to participate. Rheumatologists who are willing to participate may be more motivated individuals who are willing to take part in data collection; it is also possible that rheumatologists who conduct more tests and assessments with their patients have less time to complete surveys and take part in studies such as this. Furthermore, while DAS28(3)-ESR was selected based on data availability, the authors recognize this measure is used less frequently than DAS28(4)-ESR and does not incorporate any patient-reported measures. It is also less stringent than CDAI, SDAI, or Boolean remission [24, 33, 34]. A number of patients could not be included in the DAS28(3)-ESR analysis because of missing TJC, SJC, and/or ESR data. However, when a comparison was made between characteristics of patients who had been included in the study vs those who had been excluded due to missing data, only severity at diagnosis was shown to differ significantly between the groups, with included patients being more severe at diagnosis (Additional file 1: Table S1). The recent emergence of stricter cut-off points, not utilized in the present study which was conducted prior to their adoption in the clinical practice, leaves open the opportunity to validate current findings in future work. As this is an analysis of retrospective data, only associations between factors rather than direct causality can be assessed.

In summary, our results, consistent with published reports [35] suggests that the assessment of RA patients using standardized measures combined with protocol-specific treatment adjustments may be associated with better outcomes than patient management without use of such measures.

## Conclusions

The findings of the present study suggest that use of standardized measures of RA disease activity is not common among the rheumatologists who completed the DSP in the US. Over half of the rheumatologists reported using their own perception in assessing remission, and compared with remission assessed using standardized measures, rheumatologists were negatively discordant in the assessment of remission in nearly one-third of patients, particularly those with longstanding RA. Increasing the use of validated measures during the clinical evaluation of the RA patient may better inform treatment decisions, reduce variability in delivery of patient care, and in combination with protocol-specific treatment adjustments, may ultimately improve RA patient outcomes.

## Additional files

Additional file 1: **Table S1.** Patient demographics and clinical characteristics by inclusion in analysis. (DOCX 16 kb)

## Data Availability

Data are owned by Adelphi Real World. All requests regarding data should be addressed directly to Adelphi Real World.

## Abbreviations

ACR: American College of Rheumatology
bDMARD: Biologic disease modifying anti-rheumatic drug
BMI: Body mass index
CDAI: Clinical Disease Activity Index
CI: Confidence interval
DAS28: Disease Activity Score in 28 joints
DAS28(3)-ESR: Disease Activity Score in 28 joints (3)-erythrocyte sedimentation rate
DSP: Disease Specific Programme
EGA: Evaluator Global Assessment
EULAR: European League Against Rheumatism
HAQ-DI: Health Assessment Questionnaire-Disability Index
OR: Odds ratio
PAS: Patient Activity Scale
PGA: Patient Global Assessment
RA: Rheumatoid arthritis
RAPID-3: Routine Assessment of Patient Index Data
SDAI: Simplified Disease Activity Index
SJC: Swollen Joint Count
T2T: Treat-to-target
TJC: Tender Joint Count

## Footnotes

1 Change in pain was assessed via the question: “Please give your overall assessment of the pain that this patient experiences as a result of their RA” with pain rated on a 1–10 scale (1-None to 10- worst possible). Change in pain was then calculated as the simple difference between worse ever experienced to current – positive values indicative of an improvement.
