# Supplementary material for: The prevalence and types of discordance between physician perception and objective data from standardized measures of rheumatoid arthritis disease activity in real-world clinical practice in the US
Source: BMC Rheumatol. 2019 Jul 4;3:25. doi: 10.1186/s41927-019-0073-8 (PMC6610934; doi:10.1186/s41927-019-0073-8)
Supplement: Supplementary file 1 — Table S1. Patient demographics and clinical characteristics by inclusion in analysis. (DOCX 16 kb) [file 41927_2019_73_MOESM1_ESM.docx]

**Supplementary Table 1** Patient demographics and clinical characteristics by inclusion in analysis

| Characteristic | Overall (n = 843) | Not included (n = 312) | Included (n = 531) |
| --- | --- | --- | --- |
| **Age, in years, mean (SD)** | 56.4 (15.4) | 56.4 (15.2) | 56.4 (15.5) |
| **Female gender, n (%)** | 621 (73.7) | 224 (71.8) | 397 (74.8) |
| **BMI, mean (SD)** | 27.8 (5.9) | 27.6 (5.1) | 28.0 (6.3) |
| **Current level of disease severity, n (%)** |  |  |  |
| Mild | 612 (72.6) | 215 (68.9) | 397 (74.8) |
| Moderate/severe | 231 (27.4) | 97 (31.1) | 134 (25.2) |
| **Level of disease severity at diagnosis, n (%)*** |  |  |  |
| Mild | 586 (69.5) | 233 (74.7) | 353 (66.5) |
| Moderate/severe | 257 (30.5) | 79 (25.3) | 178 (33.5) |
| **Years since diagnosis of RA, mean (SD)** | 7.1 (7.6) | 6.3 (6.4) | 7.6 (8.1) |
| **Most recent tender joint count out of 28, mean (SD)** | n = 208 missing | n = 208 missing | n = 0 missing |
|  | 4.0 (4.9) | 3.7 (4.2) | 4.1 (5.0) |
| **Most recent swollen joint count out of 28, mean (SD)** | n = 198 missing | n = 198 | n = 0 missing |
|  | 2.9 (4.3) | 2.9 (4.2) | 2.9 (4.3) |
| **Most recent ESR (mm/hr) result, mean (SD)** | n = 172 missing | n = 172 | n = 0 missing |
|  | 25.4 (20.3) | 25.9 (18.6) | 25.2 (20.7) |
| **Patient in remission per DAS28(3)-ESR, n (%)** |  |  |  |
| No | 440 (52.2) | 173 (55.4) | 267 (50.3) |
| Yes | 403 (47.8) | 139 (44.6) | 264 (49.7) |

**P* < 0.05 [Included versus Not included]

BMI, body mass index; DAS28(3)-ESR, Disease Activity Score in 28 joints (3)-erythrocyte sedimentation rate; RA, rheumatoid arthritis; SD, standard deviation
